# The social profitability of photovoltaics in Germany

**DOI:** 10.1002/pip.2988

**Published:** 2018-02-02

**Authors:** Javier López Prol, Karl W. Steininger

**Affiliations:** ^1^ Wegener Center for Climate and Global Change University of Graz Brandhofgasse 5, A‐8010 Graz Austria; ^2^ Department of Economics University of Graz Universitätsstrasse 15, A‐8010 Graz Austria

**Keywords:** climate change, competitiveness, cost‐benefit analysis, energy transition, levelized cost of electricity, PV

## Abstract

While Germany has led the market in photovoltaic (PV) implementation throughout the last decade, there has been increasing criticism of PV support policies due to their high cost. Although declining, the levelized cost of electricity (LCOE) from PV is still above the German wholesale electricity price. However, using LCOE as an evaluation yardstick falls short in at least 2 respects: It neither takes into account integration costs rising with PV penetration (ie, undervaluing its actual cost) nor avoided externalities of replacing conventional for renewable generation (social cost overvaluation). We thus calculate the social profitability of PV in Germany by including not only private costs and benefits but also integration costs to the electricity system and avoided environmental externalities, using the internal rate of return and the profitability index as indicators. Our results show that when these factors are considered, the social profitability of PV in Germany is higher than 10% at the lower bound of the social cost of carbon (150€/tCO_2_) up to a penetration level of at least 15% and positive up to a penetration level of at least 25%. Results also show the level of private profitability if all externalities were internalized and assert that subsidies are justified to align private and social profitability. The proposed method could be used as a complementary indicator to private profitability by public institutions, development banks, and companies with social responsibility values.

## 1 INTRODUCTION

Germany has led the photovoltaics (PV) implementation market throughout the last decade, with the highest installed capacity per capita and the second highest in absolute value after China.^1^ However, although PV costs have been declining during the last 3 decades at a learning rate (ie, cost decline per each doubling in installed capacity) between 20% and 24%,^2^, ^3^, ^4^ PV support policies have been under increasing criticism due to their high costs.

While PV is quickly achieving grid parity in many parts of the world,^5^ its levelized cost (LCOE) is still above wholesale electricity prices in Germany.^4^ Additionally, increasing PV penetration causes rising integration costs to the electricity system—due to its variability, uncertainty, and location specificity.^6^, ^7^ Finally, all generation technologies also cause external costs not included in their market prices,^8^ causing social damages, which are usually ignored in traditional cost‐benefit analyses. Therefore, any comprehensive cost‐benefit analysis of generation technologies should take into account not only private costs and benefits but also the integration costs caused to the electricity system and the avoided or additional environmental externalities of switching between technologies.

We develop a method to calculate the “social profitability” of PV by including integration costs and avoided externalities alongside the assessment of private costs and benefits, applying our approach to Germany. By including social costs and benefits, we perform a more comprehensive analysis to inform policy and investment decisions. First, our method demonstrates the competitiveness of the technology when social costs and benefits are taken into account. Second, it can justify taxes/subsidies when social profitability is lower/higher than private profitability to arrive at welfare‐optimal investment decisions. Third, it indicates private profitability levels if all externalities were to be internalized. Finally, it is a useful indicator to complement private profitability for investment decisions of public institutions, development banks, and companies with social responsibility values.

We calculate “social profitability” through 2 widely used indicators: the internal rate of return (IRR) and the profitability index (PI), and according to the 2 approaches available in the literature: considering integration costs either as an additional cost or as a lower value of PV electricity.^7^ Aside from this calculation, we present a wide range of results regarding the main parameters to cope with the uncertainty concerning actual values of these parameters and their future evolution.

The remaining of the paper is structured as follows: Section 2 frames the discussion regarding the profitability and competitiveness of variable renewables. Section 3 presents the method for the calculation of the social profitability of PV and the input data to the different approaches. Section 4 presents the main results, first as a function of the social cost of carbon (SCC) and then, as a function of PV installation cost and electricity yield. Section 5 summarizes insights derived from the previous sections' analysis and its relevance.

## 2 PROFITABILITY AND COMPETITIVENESS OF VARIABLE RENEWABLES

Competitiveness of different electricity‐generating technologies is usually assessed and compared through the LCOE, which measures the life cycle costs of a technology per kilowatt hour of electricity generated during the lifetime of the system, discounted at a determined discount rate.^9^, ^10^, ^11^ However, this indicator has increasingly been criticized for its inability to capture a range of features divergent across technologies, and other indicators have been proposed, such as the levelized avoided cost of electricity, which accounts for how much it would cost the grid to generate the electricity otherwise displaced by the new generation project,^12^ and the System LCOE, which includes the cost of integrating the new generation into the existing electricity system, moving beyond the traditional LCOE.^6^

From a levelized costs perspective, competitiveness or “grid parity” is achieved when a generation technology reaches the costs of conventional technologies.^5^, ^13^ Equivalently, competitiveness can be assessed from the point of view of profitability. In this sense, competitiveness would be achieved when a technology is profitable in the market without subsidies.^14^, ^15^ However, the existence of market failures may entail the departure of private profitability from social profitability, which would not be the case in the presence of perfect and complete markets. Only in the latter case would private and social profitability be equal and the amount of investment be welfare optimal. In the presence of incomplete and/or imperfect markets, however, private investment decisions may yield socially suboptimal outcomes. In this case, the government can subsidize/tax activities with positive/negative externalities such that private and social profitability align again.^16^

Photovoltaic profitability has been widely studied regarding evaluating the impact and evolution of feed‐in tariffs and other incentives.^17^, ^18^, ^19^, ^20^, ^21^, ^22^ However, on the one hand, private profitability does not capture the integration costs caused to the electricity system in situations with higher PV penetration and, on the other hand, the avoided environmental externalities when PV displaces conventional generation. On this basis, we calculate the social profitability of PV by taking into account not only private costs and benefits but also integration costs imposed on the electricity system due to higher PV penetration as well as net avoided environmental external costs. Since we want to evaluate the social profitability of the technology to displace conventional generation, we do not include corrective incentives such as feed‐in tariffs or investment subsidies in our calculations, since they are interventions designed to correct for externalities themselves and would therefore result in double counting. Positive social profitability would entail that the technology is competitive when all factors are accounted for. Likewise, social profitability above/below private profitability would justify subsidies/taxes to that technology to adjust for social benefits/costs. Finally, social profitability shows how much private profitability would be if all externalities were internalized.

## 3 METHOD AND DATA

### 3.1 Social profitability index and social rate of return

We calculate social profitability by computing the internal rate of return and its equivalent profitability index, including not only private costs and benefits but also integration costs and avoided environmental externalities. We assume that new PV generation displaces nonrenewable generation, and we do not consider other spill‐over effects or broader macro policy objectives such as energy security or job creation.

The internal rate of return is the discount rate (*d*[%]) at which the net present value (*NPV*[€]) equals 0. The NPV is the sum of the discounted cash flows (costs and benefits) triggered by the investment during its lifetime (*n*). The first addend of Equation (1) represents the benefits derived from the electricity generated (annual electricity generated per kilowatt peak *EPV*[kWh ⋅ y^−1^ ⋅ kWp^−1^] multiplied by the wholesale electricity price *P*[€/kWh] and the value factor of PV electricity at each penetration level (*VF*_*ρ*_[proportion]). Since—as penetration (*ρ*[%]) increases—the economic effect of the variability, uncertainty, and location‐specificity of PV generation are captured as either a lower value of the electricity generated (value approach; *VF*_*ρ*_) or equivalently as a higher integration cost (cost approach; *IC*_*ρ*_[€/kWh]) at the aggregated level, we do not need high temporal resolution of PV generation and wholesale electricity prices. This simple model allows us to compute social profitability through one of the two approaches by either setting integration costs to 0 and adjusting the value factor (in the value approach) or choosing a constant value factor of 1 and acknowledging integration cost (cost approach).

*PV*_*in*_[€/kWp] and *PV*_*om*_[€ ⋅ y^−1^] are the private costs of the system: installation and operation and maintenance, respectively. Due to the quantitative importance and uncertainty related to climate change (CC), we differentiate between CC externalities (avoided carbon emissions multiplied by the respective social cost of carbon (SCC), *ACE*[tCO_2_e/kWh]^*^SCC[€/tCO_2_e], and all other avoided externalities, *EXT*[€/kWh]). Finally, *IC*_*ρ*_[€/kWh] represents the integration costs for each penetration level. The parameter *∂*_*x*_ (Equation (2)) represents the discount factor for each of the addends (*a* through *e*, see Equations (3) to (7)), capturing the annual escalation rates of each of the variables we are concerned with (*ε*_*y*_[%]), the annual rate at which the PV system loses efficiency (*dg*[%]), and the discount rate (*d*).

(1) $$\mathrm{NPV}\left(d\right)={\mathrm{EPV}}^{*}P^{*}{{\mathrm{VF}}_{\rho }}^{*}\partial_{a}-{\mathrm{PV}}_{\text{in}}-{{\mathrm{PV}}_{\mathrm{om}}}^{*}\partial_{b}+{\mathrm{EPV}}^{*}{\mathrm{ACE}}^{*}{\mathrm{SCC}}^{*}\partial_{c}+{\mathrm{EPV}}^{*}{\mathrm{EXT}}^{*}\partial_{d}-{\mathrm{EPV}}^{*}{{\mathrm{IC}}_{\rho }}^{*}\partial_{e}=0,$$

(2) $$\partial_{x}=\frac{{K_{x}}^{*}\left(1-{K_{x}}^{n}\right)}{1-K_{x}},$$

(3) $$K_{a}=\frac{\left(1+\varepsilon_{P}\right)*\left(1+\varepsilon_{\mathrm{VF}}\right)*\left(1-\mathrm{dg}\right)}{1+d},$$

(4) $$K_{b}=\frac{\left(1+\varepsilon_{{\mathrm{PV}}_{\mathrm{om}}}\right)}{1+d},$$

(5) $$K_{c}=\frac{\left(1+\varepsilon_{\mathrm{ACE}}\right)*\left(1+\varepsilon_{\mathrm{SCC}}\right)*\left(1-\mathrm{dg}\right)}{1+d},$$

(6) $$K_{d}=\frac{\left(1+\varepsilon_{\mathrm{EXT}}\right)*\left(1-\mathrm{dg}\right)}{1+d},$$

(7) $$K_{e}=\frac{\left(1+\varepsilon_{\mathrm{IC}}\right)*\left(1-\mathrm{dg}\right)}{1+d}.$$

We calculate the internal rate of return (*IRR*) as the discount rate at which the net present value (NPV) equals 0. Likewise, we calculate the PI [*PI*(*d*)] as the ratio between the NPV at a determined discount rate [*NPV*(*d*)] and the investment cost^23^, ^24^ (*PV*_*in*_; Equation (8)). The social rate of return (SRR) therefore represents the annual social yield of a PV investment at a certain penetration level, and the social profitability index [*SPI*(*d*)] represents the present value (at a predetermined discount rate) of the investment as a proportion of the investment cost. Both indicators are equivalent such that a breakeven 0% IRR corresponds to a PI of 0 at zero discount rate. Likewise, 2 *SPI*(0) is equivalent to 10% SRR (see Figure A1 in the supplementary materials for a detailed equivalence between both indicators and across different discount rates).

(8) $$\mathrm{PI}\left(d\right)=\frac{\mathrm{NPV}\left(d\right)}{{\mathrm{PV}}_{\text{in}}}$$

Finally, we can calculate the breakeven SCC, breakeven installation cost (*PV*_*in*_) and breakeven annual electricity yield (or equivalent solar irradiation) (EPV) at a certain discount rate by simply clearing the interest parameter in Equation (1) as shown, respectively, in Equations (9) to (11). In other words, we calculate the value of *SCC*, installation cost, and annual electricity yield necessary to achieve a certain level of social profitability (eg, 0%, 5%, or 10% as shown in Figures 4, 5, 6).

(9) $$\mathrm{SCC}\left(d\right)=\frac{{\mathrm{PV}}_{\text{in}}+{{\mathrm{PV}}_{\mathrm{om}}}^{*}\partial_{b}+{\mathrm{EPV}}^{*}{{\mathrm{IC}}_{\rho }}^{*}\partial_{e}-{\mathrm{EPV}}^{*}P^{*}{{\mathrm{VF}}_{\rho }}^{*}\partial_{a}-{\mathrm{EPV}}^{*}{\mathrm{EXT}}^{*}\partial_{d}}{{\mathrm{EPV}}^{*}{\mathrm{ACE}}^{*}\partial_{c}},$$

(10) $${\mathrm{PV}}_{\text{in}}\left(d\right)={\mathrm{EPV}}^{*}P^{*}{{\mathrm{VF}}_{\rho }}^{*}\partial_{a}-{{\mathrm{PV}}_{\mathrm{om}}}^{*}\partial_{b}+{\mathrm{EPV}}^{*}{\mathrm{ACE}}^{*}{\mathrm{SCC}}^{*}\partial_{c}+{\mathrm{EPV}}^{*}{\mathrm{EXT}}^{*}\partial_{d}-{\mathrm{EPV}}^{*}{{\mathrm{IC}}_{\rho }}^{*}\partial_{e},$$

(11) $$\mathrm{EPV}\left(d\right)=\frac{{\mathrm{PV}}_{\text{in}}+{{\mathrm{PV}}_{\mathrm{om}}}^{*}\partial_{b}}{P^{*}{{\mathrm{VF}}_{\rho }}^{*}\partial_{a}+{\mathrm{ACE}}^{*}{\mathrm{SCC}}^{*}\partial_{c}+{\mathrm{EXT}}^{*}\partial_{d}-{{\mathrm{IC}}_{\rho }}^{*}{\partial_{e}}^{*}}.$$

### 3.2 Cost vs value approach to integration costs

Increasing the penetration of variable renewable energy (VRE) technologies causes integration costs to the electricity system.^25^, ^26^, ^27^ Integration costs arise because of the uncertainty, variability, and location specificity of this type of technologies, which are usually higher than those of conventional dispatchable generation alternatives. Integration costs can be measured and conceptualized in a “cost approach” as the marginal cost of increasing VRE penetration or in a “value approach” as the declining marginal value of VRE electricity at higher VRE penetration.^7^ We use both approaches to calculate the social profitability of PV in Germany.

Integration costs can be decomposed into 3 subcategories according to the respective feature of the VRE causing them.^6^, ^7^ The first, *balancing costs*, arise from the uncertainty regarding the amount of potential electricity generation at any given time, with the latter depending on climatic conditions, known ahead of time with only some degree of certainty. *Grid costs* are derived likewise from the locational specificity of VRE, which must be deployed where the resource is available and the electricity then transmitted and distributed to consumption points, which entails costs related to grid use and expansion. Finally, *profile costs* are caused by the generation profile (including daily and seasonal cycles) not perfectly coinciding with load profiles and can be likewise divided into 3 subcategories: (1) backup costs derived from the low‐capacity credit of VRE, ie, the need to have idle capacity to cover the intermittency and seasonality of VRE; (2) full load hour reduction referring to the decrease of operation hours of conventional generators, which increases their cost per kilowatt hour; and (3) overproduction costs arising when generation at a given time is higher than demand. This is particularly relevant for PV as its generation is rather concentrated in a few hours of the day and months of the year.

**Figure 1:**
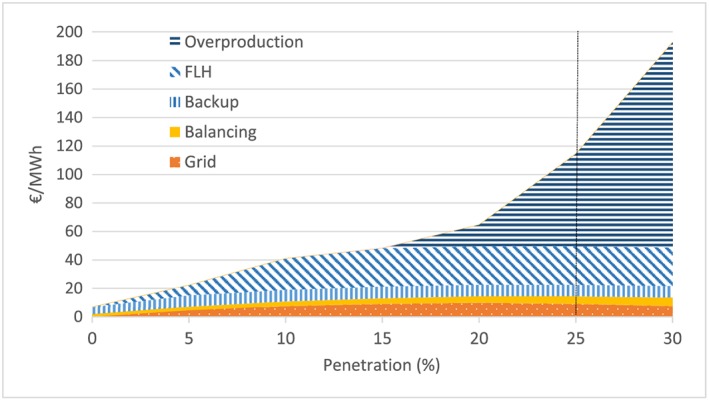
Photovoltaic integration costs across market penetration levels. Sources: Ueckerdt et al, Hirth et al, Luoma et al, Gowrisankaran et al, and MIT,^6^, ^7^, ^28^, ^29^, ^30^ as referenced by respective content in detail in the text. Note: Profile costs are extrapolated beyond 25% penetration (see Figure A4 in the supplementary materials for further details) [Colour figure can be viewed at http://wileyonlinelibrary.com]

While there is a rich literature regarding the quantification of integration costs for wind (see Ueckerdt et al and Hirth et al^6^, ^7^ for an overview), the literature on PV integration costs estimates is more scarce. The input parameters of our model—and therefore our results—are a rough estimation of shape and order of magnitude at different penetration rates, rather than a final and exact calculation. Input data are extracted from the literature and refers specifically to PV in European (profile and grid costs) and American (balancing costs) thermal systems. We assume balancing costs to be a linear function of penetration starting from 2€/MWh at 0%^28^ up to 6€/MWh at 30% penetration.^29^ According to Hirth et al,^7^ grid costs are within the single‐digit range in terms of € per megawatt hour. Therefore, we consider the most pessimistic shape of grid costs suggested by MIT^30^ (an inverted U curve peaking at 20% penetration) at an assumed 10€/MWh to be in the high end of the range. Profile costs, derived from the variability of the PV generation, entail the most important share of integration costs (Figure 1). Within profile costs, backup costs are most prominent at low penetration levels, reduction of load hours for conventional generators prevails at penetrations of 5% to 20%, and overproduction costs rocket to dominance beyond that point.^6^ Figure 1 illustrates the input data for the cost model, which is derived from the aforementioned literature and constitutes a quantification of the shape and order of magnitude rather than an exact calculation.^6^

Likewise, increasing penetration of almost‐zero marginal cost electricity technologies cause the decline of wholesale electricity prices by shifting the supply curve to the right in the so‐called *merit‐order effect*.^31^, ^32^, ^33^, ^34^ This effect is particularly relevant for PV since the price drop is stronger at times of high PV generation. Therefore, the market value of PV electricity declines as its penetration in the market increases.^35^, ^36^, ^37^, ^38^, ^39^ In the presence of perfect and complete markets, the decline in the value of PV electricity would be equal to the decrease of its value factor in the wholesale electricity market, where the value factor represents the ratio of the unit revenue of PV (ie, the generation‐weighted average price) and the time‐weighted average wholesale electricity price,^39^, ^40^ in other words, the average remuneration of PV producers relative to the average wholesale price. Therefore, integration costs can also be understood as the decline in the market value of PV electricity, this approach being equivalent to the cost perspective.^6^, ^7^

**Figure 2:**
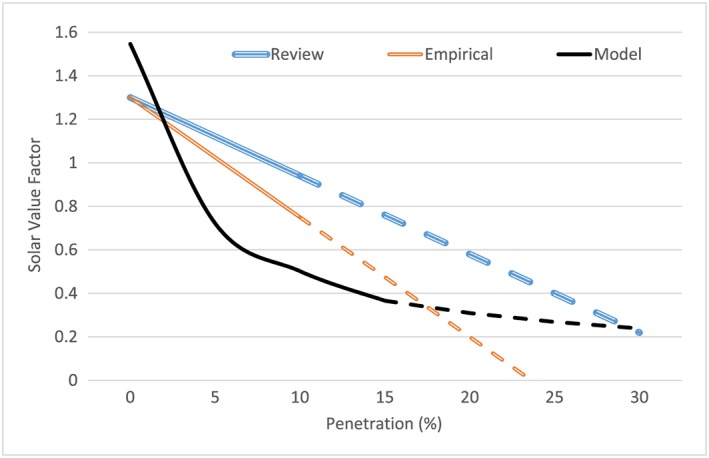
Solar value factor trends across penetration levels according to literature estimates. Note: both “review” and “empirical” estimates are derived from ex‐post analysis of the wholesale electricity market for different countries (review) and for Germany (empirical), while “model” estimates come from an electricity system investment and dispatch model. Dashed segment of the lines are extrapolations. See sources^39^, ^40^ for a more detailed illustration and Figure A4 in the supplementary materials for more details on the extrapolations [Colour figure can be viewed at http://wileyonlinelibrary.com]

The market value of PV electricity can be estimated empirically ex‐post from market data or theoretically ex‐ante through dispatch or investment and dispatch models. Hirth^39^, ^40^ provides a comprehensive overview of the concept and estimations of the market value of PV electricity. First, he reviews estimates found in the literature for different countries and penetration levels. Second, he estimates ex‐post value factors from the German wholesale electricity market. Finally, he runs the European Electricity Market Model (EMMA) dispatch and investment model to estimate the ex‐ante long‐term value factors for penetration levels up to 15%. Their average results are illustrated in Figure 2 (labelled as “review,” “empirical,” and “model”), with respective extrapolations (linear for the review and empirical, and power for the model estimates) up to 30% penetration.

Figure 2 shows high variability of the value factor estimates across penetration levels between the different estimations.^39^, ^40^ The difference between the 2 ex‐post estimations (review and empirical) derives from the fact that the review regression line is the result of all the estimates found in the literature about different countries and their respective penetration levels, while the empirical refers only to the German electricity market. Among these 2, the empirical would be more suitable for our analysis than the review estimates as it relates to the country of our concern. However, only the ex‐ante approach allows for the flexibility necessary to accommodate future changes in the electricity mix. While the ex‐post approach relies on an extrapolation of the current electricity system, in the investment and dispatch model, the electricity system adapts capacity and generation to higher penetration of variable renewables. That is why we use the results of the latter, the ex‐ante investment and dispatch model, as input parameters for the calculation of the social rate of return.

We use the model benchmark estimates at 0€/tCO_2_ SCC (since we account for the SCC separately) and its respective power extrapolation (see Figure A4 in the supplementary materials for more details on extrapolations) as input data. The estimates at 0€/tCO_2_ show a higher solar value at low penetration but a stronger decline as penetration increases. These estimates are derived from the partial equilibrium EMMA model, which is a dispatch and investment model representing the Northwestern European power system (Germany, Belgium, The Netherlands, France, and Poland) and does not account for internal grid constraints, so it only partially captures grid integration costs, the total of which are relatively small according to Ueckerdt et al^6^ (see Figure 1).

In conclusion, both cost and value approaches are suitable and theoretically equivalent to account for the integration costs of PV or any other VRE. However, the input data for both approaches are estimated differently, which explains the differences in our results for each of the approaches. The cost approach input data is less consistent, since the different components come from different studies and are only rough estimations of shape and order of magnitude, with balancing and grid cost interpolation and profile cost extrapolation between 25% and 30% penetration. The value approach input data is more recent and consistent; however, it only partially captures grid costs, and since extrapolation starts at 15% penetration, it is unlikely to capture the overproduction costs arising from this penetration level. Although both approaches include endogenous adaptation (ie, optimal response of the electricity mix to increasing VRE penetration), the cost approach shows the “worst‐case scenario” without any additional adaptation, while the value approach, applied beyond 15% penetration, shows the “best case” if all overproduction costs could be avoided with additional adaptation measures such as storage, demand side adjustment, or intercontinental interconnections.†

Our model allows for the computation of both cost and value approaches. Table 1 summarizes the input values for each of the approaches, taking into account that integration costs would be 0 in the value approach, and likewise, the value factor would be 1 in the cost approach. We calculate our model in real terms and assume that the real integration costs and value factor are unchanging because of reasons other than the market penetration level.‡

**Table 1:** Input parameters for the cost and value approach (see Figures 1 and 2, respectively)

Penetration, [%]	Integration Cost, *IC*[€/MWh]	Value Factor, *VF*[PV unit revenue/avg. wholesale price]
0	7	1.546
5	22.3	0.722
10	41.0	0.502
15	48.2	0.366
20	64.7	0.258*
25	114.6	0.184*
30	192.7*	0.131*
Source	Ueckerdt et al, Hirth et al, Luoma et al, Gowrisankaran et al, and MIT^6^, ^7^, ^28^, ^29^, ^30^	Hirt 2013 and 2015^39^, ^40^

Abbreviation: PV, photovoltaic.

* Extrapolation (see Figure A4 in the supplementary materials for details).

### 3.3 Climate change and other environmental externalities

Externalities can be defined as the costs or benefits caused by an economic activity to a third party not involved in the transaction. Since external costs or benefits are not included in market prices, they lead to a welfare‐suboptimal allocation of resources.^16^ In the case of energy, virtually all generation technologies impose external costs not included in their price, such as human toxicity, depletion of resources, or climate change (CC), among others.^8^ Reaching a welfare‐optimal allocation of resources requires the integration of externalities into market processes by, for instance, taxing/subsidizing activities with negative/positive externalities.

**Figure 3:**
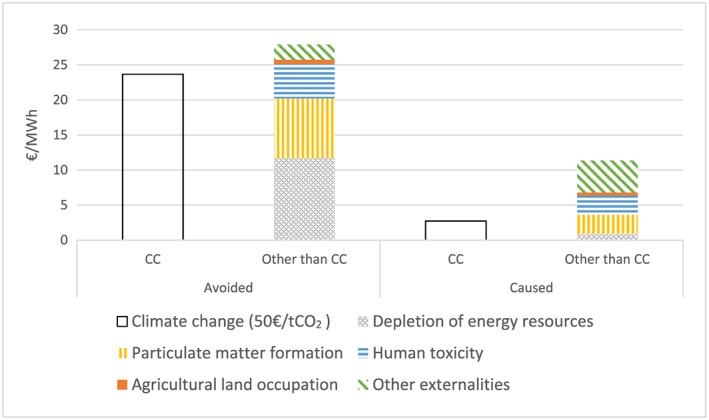
Average external cost per megawatt hour of the nonrenewable German electricity generation (avoided) and of photovoltaic (PV, caused). Sources: Ecofys^8^ and Eurostat (nonrenewable generation). For illustrative purposes, the figure depicts climate change external costs at 50€/tCO2 social cost of carbon. In Section 4, we study a range of the social cost of carbon between 0 and 300. CC, climate change [Colour figure can be viewed at http://wileyonlinelibrary.com]

Due to the complexity and uncertainty regarding the CC externality caused by the release of CO_2_ and other greenhouse gases (GHG), externalities are divided in our model into CC externalities, valued at the SCC, and other externalities, for which we use the estimates provided by Ecofys.^8^ We include the net positive externalities of PV electricity by subtracting the negative externalities of PV itself from the per kilowatt hour external cost of nonrenewable German electricity generation that PV substitutes for, as shown in Figure 3.

Since we assume that the new PV generation displaces conventional generation, the avoided carbon‐equivalent emissions are given by the CO_2_‐equivalent emissions intensity of nonrenewable generation in Germany, provided by Eurostat and valued at the SCC. The SCC is defined as the marginal cost of carbon dioxide‐equivalent emissions caused by CC damages with respect to a business‐as‐usual scenario. The SCC, estimated through integrated assessment models (IAMs), is uncertain. The most comprehensive review of the SCC estimates has been done by the US Interagency Working Group, which analyzes the 3 main models [Policy Analysis of the Greenhouse Effect (PAGE), Climate Framework for Uncertainty, Negotiation and Distribution (FUND), and Dynamic Integrated Climate‐Economy model (DICE)] and the 5 most relevant scenarios to reach a central estimate^41^ of 36$_2007_/tCO_2_ in 2015. Another review of more than 200 estimates^42^ determines an average value§ of 41$_1995_/tCO_2,_ and the most recent update of the DICE model suggests a value^43^ of 31$_2010_/tCO_2._

These average estimates, however, conceal large uncertainties. For instance, Ackerman and Stanton^44^ show the large range of estimates provided by the DICE model by simply exploring the plausible values of 4 types of uncertainties: (1) climate sensitivity, climate damages at (2) low and (3) high temperatures, and (4) the social discount rate, obtaining values between 28$/tCO_2_ and 892$/tCO_2_ in 2010. Likewise, Pindyck criticizes the pretension of knowledge provided by IAMs and argues that climate policies should not be based on average outcomes but on the possibility of extreme events, and urges the implementation of carbon prices to internalize the CC externality.^45^, ^46^, ^47^, ^48^

In this sense, van den Bergh and Botzen^49^ argue that the average estimates from IAMs are gross underestimates: first, because they include both low and high social discount rates, which undermine the present value of future damages, and second, because they ignore factors such as (1) uncertainty about GHG concentrations, (2) costs of a large rise in temperature, (3) overall higher climate damages, (4) low‐probability/high‐impact CC risks, and (5) risk aversion. According to this meta‐analysis, the lower bound of the SCC should be at least 125$_1995_/tCO_2_ (ie, about 150€_2015_/tCO_2_).** The fact that IAMs do not generally take into account the existence of tipping points and positive CC feedback loops, which is also likely to be a source of undervaluation, in addition to the latest developments in earth and climate sciences, which point at higher climate sensitivity than expected,^50^ permafrost tipping point risks,^51^ higher than expected sea level rise,^52^, ^53^ and even climate‐driven polar motion,^54^ lead us to expect that the SCC might be even higher than the lower bound of 150€_2015_/tCO_2_ suggested by van den Bergh and Botzen.^49^ We still use the value of 150€/tCO_2_ as a benchmark lower bound of the SCC but report a wide range between 0 and 300€/tCO_2_ to account for the uncertainty surrounding its actual value and the (more likely than not) upwards evolution of future estimations acknowledging risk aversion.

### 3.4 Other data and assumptions

Since we calculate our model in real terms and assume that nominal operation and maintenance costs, electricity prices, and integration costs (the latter over time but at identical penetration levels) rise at the same rate as inflation, their respective real escalation rates are equal to 0. The SCC increases over time because GHG concentrations increase over time, with future emissions thus producing larger incremental damages.^41^ Although its real escalation rate is also uncertain (between 1.2% and 4.4% depending on the assumptions and the timeframe^41^), we assume an intermediate value of 2.5%. We assume an installation cost of 1000€/kWp,^11^ the wholesale electricity price at its average value between years 2005 and 2010, and a system lifetime of 25 years (see Table 2 for a summary of the input data).

**Table 2:** Data and assumptions for the social profitability calculations

Parameter	Installation Cost	Operation and Maintenance Cost	Electricity Price (2005‐2010 av.)	Electricity Yield	Net Avoided Carbon Emissions	Net Avoided External Cost
Notation [unit]	*PV*_*in*_[€/kWp]	*PV*_*om*_ [€ ⋅ y^−1^]	*P*[€/*MWh*]	*EPV*[kWh ⋅ y^−1^ ⋅ kWp^−1^]	*ACE*[*tCO*_2_*e*/*MWh*]	*EXT*[€/GWh]
Value	1000	10	48.375	938*	0.701	16.54
Source	Fraunhofer^11^	Talavera^17^	Epex spot	Šúri et al^55^	Eurostat	Ecofys^8^

* With a 0.8% annual degradation rate.^56^

## 4 RESULTS AND DISCUSSION

We now present our model results, comparing the cost and the value approaches. While their interpretation up to 15% penetration is the same, it differs beyond that point. Both approaches include endogenous adaptation of the electricity system to higher VRE generation. However, while the cost approach includes overproduction costs arising beyond 15% penetration, these are not captured in the value approach, since input data are extrapolated beyond the point at which overproduction start to arise. Therefore, the cost and value illustrations below can be considered as worst and best cases, respectively, considering either no additional adaptation measures beyond endogenous electricity system capacity optimization (cost approach) or perfect adaptation to remove all overproduction costs beyond 15% penetration (value approach).

**Figure 4:**
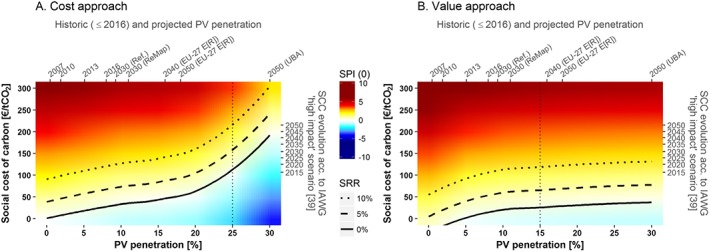
Social profitability index at 0% discount rate [*SPI*(0)] and social rate of return (SRR) as a function of PV penetration [%] and the social cost of carbon (SCC, €/tCO_2_). Note: The right‐side axis illustrates the year, in which the corresponding SCC is reached according to the “high‐impact” scenario of the Interagency Working Group.^41^ The upper axis depicts the evolution of PV penetration in Germany^57^ up to 2016, and different projections for Germany (International Renewable Energy Agency Reference and ReMap scenarios^58^ for 2030 and Umweltbundesamt [UBA] projection for the 100% renewables scenario^59^ in 2050) and the EU‐27 (Energy [R]evolution scenario^60^). Input data used for calculations to the right of the vertical dotted lines are extrapolations

**Figure 5:**
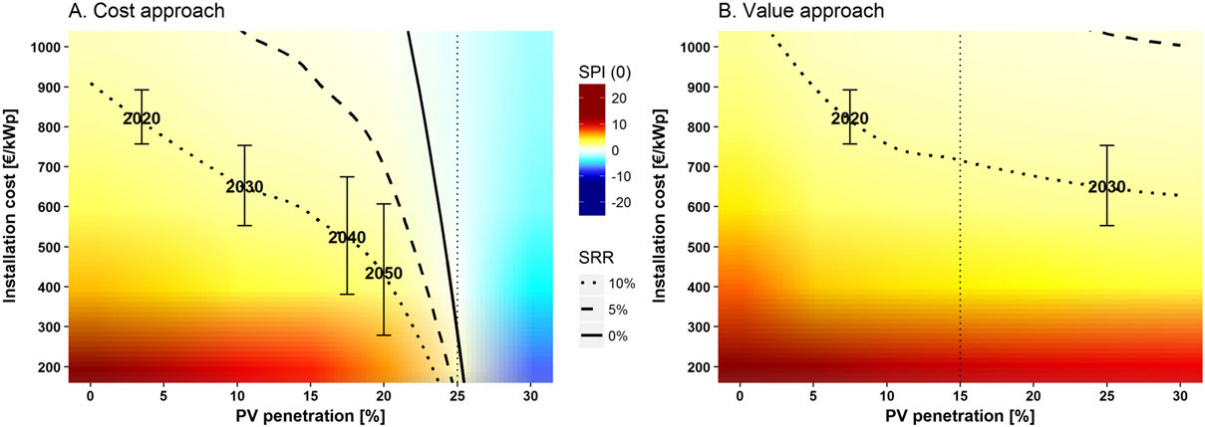
Social profitability index at 0% discount rate [*SPI*(0)] and social rate of return (SRR) at 75€/tCO_2_ social cost of carbon as a function of photovoltaic (PV) penetration (%) and installation cost (*PV*_*in*_[€/kWp]). Note: Ranges labelled by years represent the forecasted evolution of PV installation costs by the Fraunhofer Institute^11^ with their respective uncertainty ranges, arbitrarily located along the 10% SRR line for a clear visualization. Input data used for calculations to the right of the vertical dotted line are extrapolations

**Figure 6:**
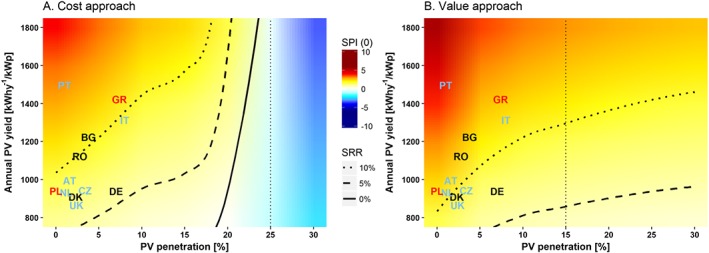
Social profitability index at 0% discount rate [*SPI*(0)] and social rate of return (SRR) at 75€/tCO_2_ social cost of carbon as a function of photovoltaic (PV) penetration [%] and annual PV yield (*EPV*[kWh ⋅ y^−1^ ⋅ kWp^−1^]). Note: Country codes written in black have ±10% of the German emission intensity of the nonrenewable generation, countries in blue have one between 10% and 50% lower, and countries in red have an emission intensity of nonrenewable generation between 10% and 50% higher. Input data used for calculations to the right of the vertical dotted line are extrapolations

The cost of capital is not included in the model so it is under the discretion of the reader to judge the required profitability level to cover the opportunity cost of capital. However, if we assume that PV deployment is financed by the government to correct electricity market externalities, it is reasonable to consider the 25‐year German bond yield as a plausible cost of capital for PV subsidies. The interest rates on 25‐year German bonds have ranged between 1% (in 2017) and 5% in the last 10 years, so the white area between the solid and the dashed lines in Figures 4, 5, 6, representing 0% and 5% social profitability levels, respectively, is a plausible range for covering the “social cost of capital.”

### 4.1 Social profitability as a function of the SCC

Figure 4 shows the results for the 2 approaches as a function of PV penetration and the SCC. The color gradient represents the social profitability index (SPI) at 0% discount rate, and the solid line shows the social profitability breakeven (ie, social rate of return (SRR) equal to 0%). The dashed and the dotted lines show SRR of 5% and 10%, respectively (see Figure A1 in the supplementary materials for a detailed relation between the SPI and the SRR and between the SPI at different discount rates). Both cost and value approaches agree that PV profitability at current penetration (8%)^57^ is above 10% at the lower bound of the SCC (150€/tCO_2_), and around 5% at a SCC as low as 50€/tCO_2_. Indeed, PV would still be socially profitable at that level of the SCC and double the current penetration (50€/tCO_2_ and 16% penetration).

While the primary axes provide the basic information to interpret Figure 4, we provide illustrative secondary axes to help the reader evaluate their own dynamic scenarios. Thus, the upper axis shows the years in which different penetration levels were achieved in Germany (eg, 8% in 2016), as well as different projections regarding the future evolution of PV penetration for Germany (International Renewable Energy Agency Reference and ReMap scenarios^58^ for 2030 and Umweltbundesamt (UBA) projection for the 100% renewables scenario^59^ in 2050) and the EU‐27 (Energy [R]evolution scenario^60^). Since the SCC increases over time (because future emissions occur at a higher GHG concentration level and thus produce larger incremental damages), the right‐side axis illustrates the years, in which the corresponding values of the SCC are reached according to the “high‐impact” (or risk acknowledging) scenario of the Interagency Working Group.^41^ This scenario considers a SCC of 107€_2015_/tCO_2_ in 2015 rising up to 215€_2015_/tCO_2_ by 2050.††

The wide range of parameters provided in Figure 4 allows the reader to evaluate their own dynamic scenarios according to the assumed value and evolution of the SCC, the expected evolution of PV penetration, and the required social profitability. For instance, if we assume a desired social profitability at 5% and the evolution of the SCC given by the high‐impact scenario of the Interagency Working Group, the dashed line in Figure 4A would represent a diffusion pathway such that the maximum socially desired PV penetration in 2015 would be 20%, 25% in 2030, and almost 30% in 2050 at current installation costs (see Section 4.2 for different installation costs).

Both cost and value approaches show similar results up to 15% PV penetration, which suggests the accuracy of the input data. Beyond that point, the cost approach represents the worst case scenario given current conditions, while the value approach would represent the potential for PV social profitability if overproduction costs could be totally removed with additional adaptation measures, such as demand side adjustment, storage, or intercontinental interconnections.

The positive social profitability of PV entails that the technology is competitive when factoring in both integration costs and the external cost of conventional generation. This can, on one hand, justify subsidies for this technology such that private profitability aligns with social profitability. On the other hand, this method could be used to compare different investment alternatives in terms of social profitability rather than only private profitability, for which more transparency and data on externalities valuation is needed to make this kind of analysis possible and accurate. Finally, it shows how profitable PV would be if all externalities were internalized.

### 4.2 Social profitability as a function of the installation cost

We now extend our analysis to cover a wide range of possibilities regarding PV installation costs and PV yield per kilowatt peak, which deepen the dynamic interpretation of the results and allow the reader to evaluate different scenarios according to assumptions on the evolution of different parameters. Since at the lower bound of the SCC (150€/tCO_2_) PV is socially profitable for the entire assessed penetration range, we present the results for half that value (75€/tCO_2_) to better depict the relationship between these variables and PV penetration (see Figures A3 and A4 in the supplementary materials for results with SCC of 0€/tCO_2_, 50€/tCO_2_, and 150€/tCO_2_).

Figure 5 shows the social profitability of PV at increasing penetration depending on the installation cost, with the SCC set at 75€/tCO_2_. Both approaches agree that (1) the lower the installation cost, the higher the spread of the social profitability across penetration levels, and (2) at the current installation costs and SCC at half its lower bound, PV penetration would be socially profitable up to 20%. Beyond that penetration level, the cost approach shows a strong and rapid decline in social profitability and a virtual penetration barrier beyond 20% to 25%. This suggests that PV will only be profitable at high penetration levels if CC damages (and therefore the SCC) are high. Exogenous adaptation, however, could substantially improve the social profitability of PV as suggested by the value approach. Figure 5 also shows the forecasted evolution of installation costs in Germany by the Fraunhofer Institute^11^ between 2020 and 2050. According to their average estimations, and in the worst case (represented by the cost approach with SCC at only half its lower bound value), PV would achieve 10% social profitability at 10% penetration in 2030 (see supplementary materials for results at different SCC values).

### 4.3 Social profitability as a function of the PV yield per watt peak

Finally, we present the social profitability of PV as a function of its penetration and its potential yield per kilowatt peak, again with a SCC at only half its lower bound (75€/tCO_2_). The potential PV yield per kilowatt peak is taken from the Photovoltaic Geographical Information System (PVGIS),^55^ which assumes a 75% performance rate. Figure 6 illustrates the position of several European countries as a function of their respective PV penetration and average PV potential. Due to the lack of country‐specific integration cost estimations, these results can provide a useful illustration about their approximate position. However, since avoided carbon emissions is a key variable, countries with ±10% of the German level are depicted in black, countries with levels between 10 and 50% lower emissions intensity of their nonrenewable generation are depicted in blue (meaning that their social PV profitability is actually lower than their position implies due to the lower abatement benefits), and countries with emissions intensity at levels between 10% and 50% higher are depicted in red. Countries beyond ±50% German levels are not depicted at all.

All selected countries show positive social profitability and a potential to increase penetration up to at least 20% at half the lower bound of the SCC. Greece and Poland, although differing on their solar irradiation and penetration level, both show PV potential due to their high emissions intensity. Portugal and Italy, although having lower potential abatement benefits due to their lower emissions intensity, have high social PV profitability, thanks to their high insolation. Social profitability of PV would be around 10% in Romania and Bulgaria, according to the conservative scenario depicted in Figure 6A.

## 5 CONCLUSIONS

We have calculated the social profitability of PV in Germany by including not only private costs and benefits but also integration costs of higher PV penetration in the electricity system and avoided external costs of displacing nonrenewable generation. We have computed social profitability from a cost and value perspective. Both approaches agree that at the lower bound of the SCC (150€/tCO_2_), PV social profitability is above 10% at current German penetration level (8%), and it is still positive up to at least double the current penetration level even at only 50€/tCO_2_.

This entails that PV is competitive when both integration costs and externalities are included in the analysis and shows the level of private profitability if all the external costs and benefits were internalized. Therefore, subsidies to PV are economically justified in these ranges to align private and social profitability and reach a welfare‐optimal allocation of resources.

Although uncertainties are large for penetration levels beyond 15%, the comparison between cost and value approaches suggests the high potential of exogenous adaptation (such as storage, demand side adjustment, and intercontinental interconnections) to boost PV social profitability also in situations beyond 20% penetration. In the worst case, if no additional adaptation measures are implemented, PV will only be competitive beyond 20% penetration to abate high CC damages at exponentially increasing costs due to overproduction. On the contrary, if all overproduction costs could be totally removed at zero cost, PV would be socially profitable at half the lower bound of the SCC, independently of its penetration.

The method presented in this article could be used as a complementary indicator to evaluate investment alternatives by public institutions, investment banks, and companies with social responsibility values. Additionally, it would be interesting to apply this method to other technologies and types of investments to be able to compare them with PV and have a more comprehensive understanding of the role of different abatement technologies. For instance, it could well be that other technologies or types of investments (eg, wind installations or efficiency measures) have higher social profitability than PV in certain circumstances. It is also important to further improve the data related to external costs as well as their estimation, so that this type of analysis can be generalized and extended to other technologies and sectors. Finally, it would also be valuable to explore different scenarios of PV social profitability depending on different types and levels of adaptation measures to cope with overproduction costs.

## Supporting information

Figure A1. Relation between indicators across profitability indexes
Figure A2. Social profitability index at 0% discount rate (*SPI*(0)) and social rate of return (SRR) as a function of the PV penetration [%] and the social cost of carbon (SCC [€/tCO_2_]). Cost approach excluding overproduction costs
Figure A3. Social profitability index at 0% discount rate (*SPI*(0)) and social rate of return (SRR) at 0, 50 and 150 €/tCO_2_ social cost of carbon as a function of PV penetration [%] and installation cost (*PV*_*in*_ [€/kWp])
Figure A4. Social profitability index at 0% discount rate (*SPI*(0)) and social rate of return (SRR) at 0, 50, and 150 €/tCO_2_ social cost of carbon as a function of PV penetration [%] and annual PV yield (*EPV*[kWh ⋅ y^−1^ ⋅ kWp^−1^]).
Figure A5. Input data extrapolations for integration costs and value factors

## ENDNOTES

†
Indeed, the value approach results beyond 15% penetration show a similar shape as produced by the cost approach when overproduction costs are excluded (see Figure A2 in the supplementary materials).

‡
More specifically, this results in a profitability index or IRR for a specific penetration level. If penetration increases further in the future, this may induce changes to the integration costs or value factor, also for all earlier investments. As we neglect this potential impact, our results are valid for the respective point in time and a specific penetration level, but profitability and SRR could decline for such investment, when penetration is further enhanced later—in the same way as the new profitability index at the higher penetration level will decline.

§
Originally reported in $ per ton of carbon^46^: 151$ per ton of carbon is equivalent to 41.2$ per ton of CO_2_.

**
van den Bergh and Botzen^53^ derive the lower bound of the SCC of 125$_1995_/tCO_2_ by applying a 206% surcharge on the average value indicated by Tol^46^ (personal communication to authors). We apply the US GDP deflator (Federal Reserve database) for 1995 to 2015 (1.46) and the 2015 average US$ to € exchange rate (1.11) and perform a sensitivity analysis with the long‐term (1990‐2015) average exchange rates (1.11‐1.23) to account for the real depreciation of the US$ in the last years, together resulting in a range between 148€_2015_/tCO_2_ and 164€_2015_/tCO_2_, out of which we consider a conservative value of 150€_2015_/tCO_2_ as a benchmark for the lower bound of the SCC.

††
Using the US GDP deflator between 2007 and 2015 (1.128) and the US$/€ average exchange rate in 2015 (1.11) provided by the Federal Reserve.
