# Supplementary material for: The social profitability of photovoltaics in Germany
Source: Prog Photovolt. 2018 Feb 2;26(8):631–41. doi: 10.1002/pip.2988 (PMC6108396; doi:10.1002/pip.2988)
Supplement: Supplementary file 1 — Figure A1. Relation between indicators across profitability indexes Figure A2. Social profitability index at 0% discount rate (SPI(0)) and social rate of return (SRR) as a function of the PV penetration [%] and the social cost of carbon (SCC [€/tCO2]). Cost approach excluding overproduction costs Figure A3. Social profitability index at 0% discount rate (SPI(0)) and social rate of return (SRR) at 0, 50 and 150 €/tCO2 social cost of carbon as a function of PV penetration [%] and installation cost (PVin [€/kWp]) Figure A4. Social profitability index at 0% discount rate (SPI(0)) and social rate of return (SRR) at 0, 50, and 150 €/tCO2 social cost of carbon as a function of PV penetration [%] and annual PV yield (EPV[kWh ⋅ y−1 ⋅ kWp−1]). Figure A5. Input data extrapolations for integration costs and value factors [file PIP-26-631-s001.docx]

**Appendix**

Figure A.1. Relation between indicators across profitability indexes


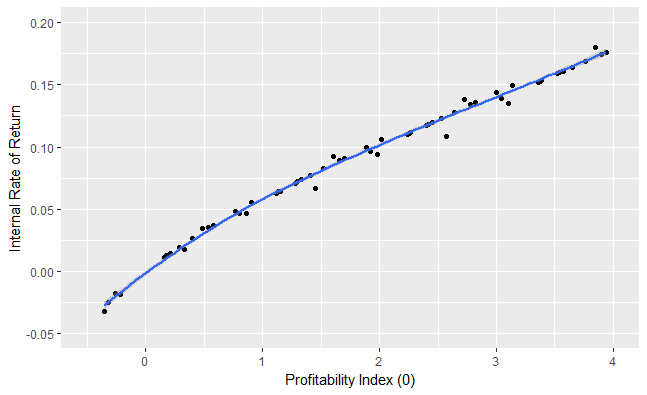
A) Relation between the profitability index at 0% discount rate and the internal rate of return

B) Correlation between the profitability index at different discount rates


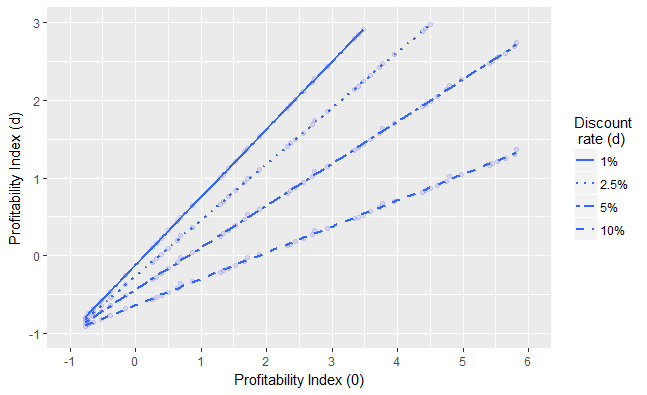


Figure A.2. Social profitability index at 0% discount rate ($SPI(0)$) and social rate of return (SRR) as a function of the PV penetration [%] and the Social Cost of Carbon (SCC [€/tCO_2_]). Cost approach excluding overproduction costs.


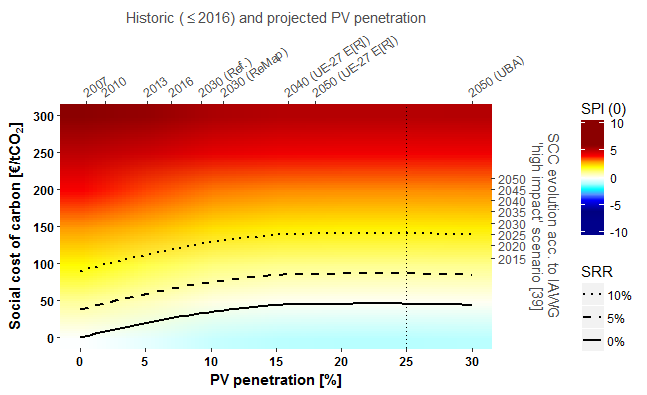


Note: the right-side axis illustrates the year in which the corresponding SCC is reached according to the “high impact” scenario of the Interagency Working Group [41]. The upper axis depicts the evolution of PV penetration in Germany up to 2016 [54], and different projections for Germany (IRENA Reference and ReMap scenarios for 2030 [55] and UBA projection for the 100% renewables scenario in 2050 [56]), and the EU-27 (Energy [R] evolution scenario [57]). Input data used for calculations to the right of the vertical dotted lines are extrapolations (see Fig. A4 in the appendix).

Figure A3. Social profitability index at 0% discount rate ($SPI(0)$) and social rate of return (SRR) at 0, 50 and 150 €/tCO_2_ social cost of carbon as a function of PV penetration [%] and installation cost ($PVin$ [€/kWp])


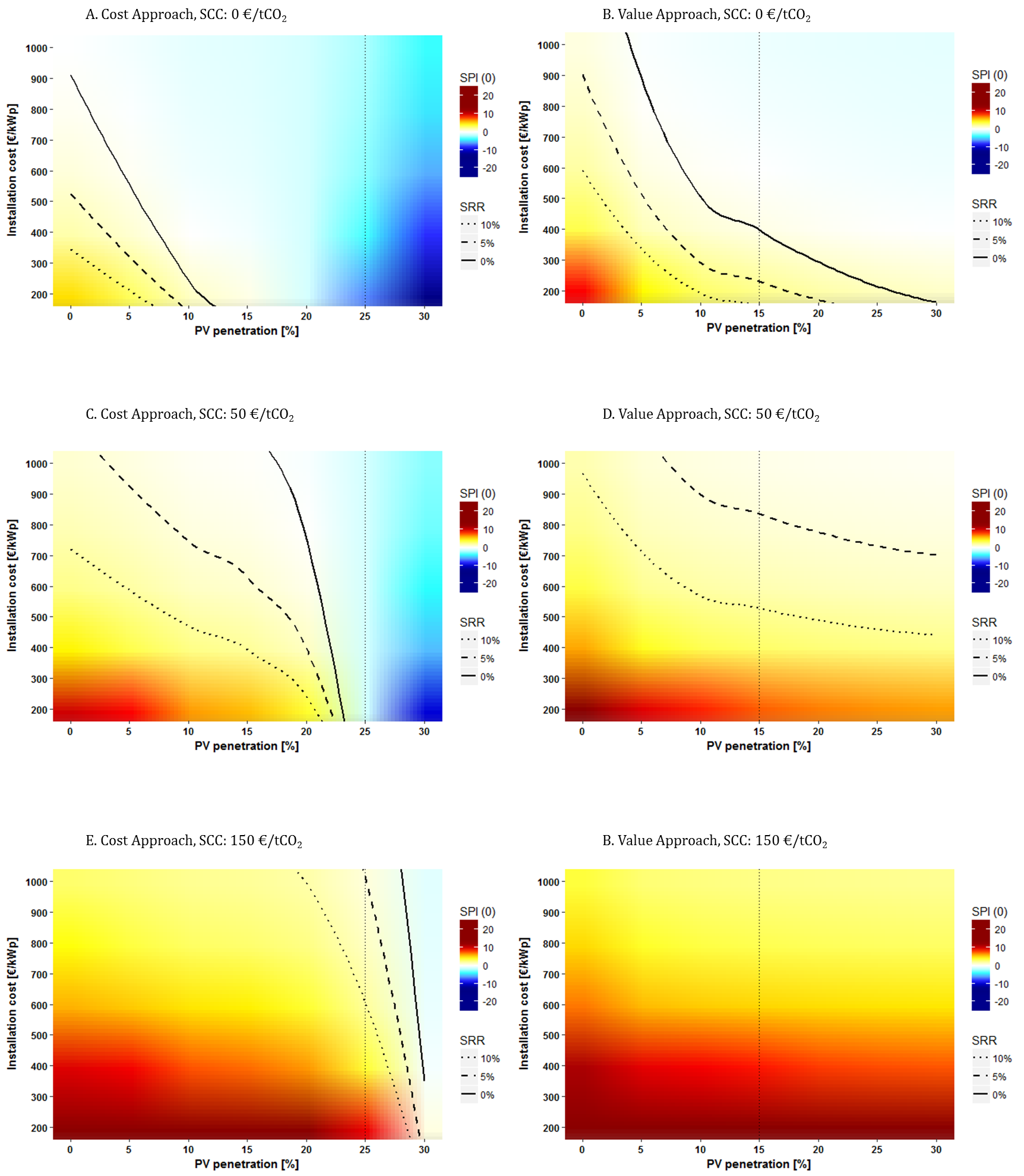


Input data used for calculations to the right of the vertical dotted line are extrapolations.

Figure A.4. Social profitability index at 0% discount rate ($SPI(0)$) and social rate of return (SRR) at 0, 50, and 150 €/tCO_2_ social cost of carbon as a function of PV penetration [%] and annual PV yield ($EPV [kWhy^{-1}/kWp]$).


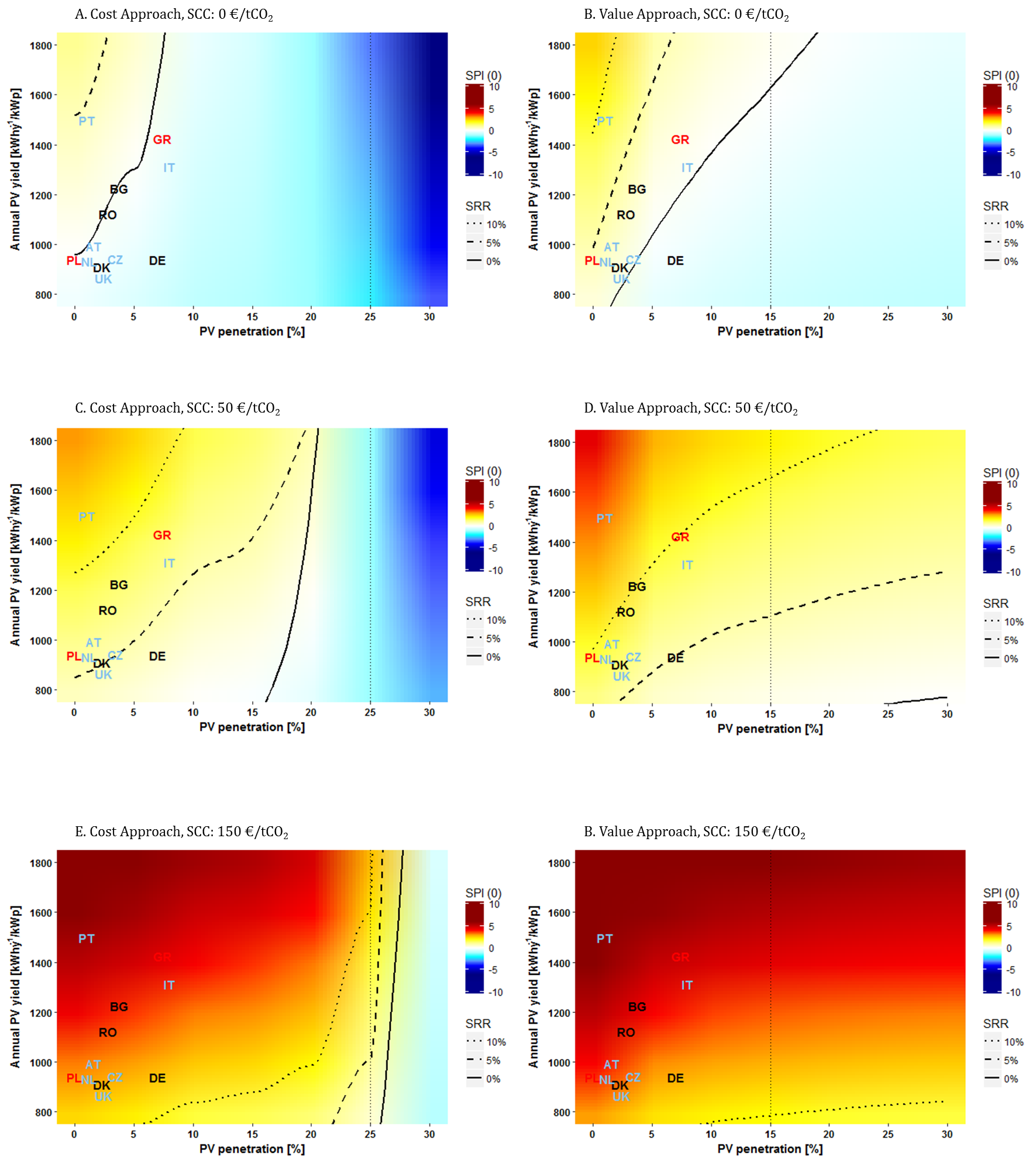


Note: country codes written in black have ± 10% of the German emission intensity of the non-renewable generation, countries in blue have one between 10-50% lower, and countries in red have an emission intensity of non-renewable generation between 10-50% higher. Input data used for calculations to the right of the vertical dotted line are extrapolations. The span of the smoothing function may differ across figures for visualization purposes.

Figure A4. Input data extrapolations for integration costs and value factors:

A) Polynomial extrapolation of integration cost

B) Power extrapolation of Value Factor
